# CircDHRS3 inhibits prostate cancer cell proliferation and metastasis through the circDHRS3/miR-421/MEIS2 axis

**DOI:** 10.1080/15592294.2023.2178802

**Published:** 2023-02-25

**Authors:** Xiyu Dai, Xinan Chen, Wensun Chen, Yuxi Ou, Yiling Chen, Siqi Wu, Quan Zhou, Chen Yang, Limin Zhang, Haowen Jiang

**Affiliations:** a Department of Urology, Huashan Hospital, Fudan University, Shanghai, China; b Fudan Institute of Urology, Huashan Hospital, Fudan University, Shanghai, China; c National Clinical Research Center for Aging and Medicine, Fudan University, Shanghai, China

**Keywords:** circDHRS3, circRNA, prostate cancer, MEIS2

## Abstract

Prostate cancer is the most prevalent type of cancer among men worldwide. The importance of circular RNA (circRNA) in prostate cancer and its connection to malignancy has been steadily recognized. circRNA expression was obtained by circRNA sequencing of prostate cancer. circRNA and its function were further analysed. The results were verified by qRT-PCR, RIP assay, FISH, RNA pulldown, WB, CCK-8, colony formation assay and wound-healing assay. BALB/c Nude mice were used for xenograft hosts. Low expression of circDHRS3 was assessed in prostate cancer. Overexpression of circDHRS3 inhibited prostate cancer growth and migration *in vitro*. Additionally, miR-421 was shown to be the downstream target of circDHRS3, as shown by fluorescence *in situ* hybridization and dual-luciferase experiments. The rescue assay results for the PC3 and Du145 cell lines demonstrated that circDHRS3 inhibits prostate cancer cell lines’ ability to proliferate and metastasize by modulating MEIS2 expression through the circDHRS3/miR-421/MEIS2 axis. *In vivo* investigations confirmed that the overexpression of circDHRS3 could inhibit both the lung and bone metastasis of prostate cancer cells. circDHRS3 has the potential to become a biomarker and a targeted therapeutic site for prostate cancer, particularly in the malignant stage. Our study indicates that circDHRS3 inhibits prostate cancer cell proliferation and metastasis through the circDHRS3/miR-421/MEIS2 axis.

## Background

Prostate cancer is the most prevalent type of cancer among men, becoming increasingly common globally in recent decades, and is the second greatest cause of cancer-related death in men [1,2]. Prostate cancer can be defined as either a localized or advanced disease, with various treatments available including surgery, radiation, androgen deprivation therapy (ADT), chemotherapy, and combinations of these therapies. Although these strategies are beneficial to individuals during various stages of prostate cancer, their efficacy is still restricted as death rates continue to increase, owing to metastasis [3]. Therefore, finding new molecular targets for diagnosing and treating prostate cancer is urgently needed.

Non-coding RNA molecules, with a single closed covalent loop, are known as circular RNA molecules (circRNAs). circRNAs lack a 5′ cap structure and a 3′ polyadenylated tail structure [4]. circRNAs possess higher stability than their linear form and can be generated by the non-classical back-splicing of mRNA to a circular configuration [5,6]. Due to the resistance of circRNAs to RNase R, they may play a role in disease diagnosis and treatment as a stable new biomarker [7]. A well-recognized ncRNA (no-coding RNA) regulation mechanism of circRNAs, further involved in regulating biological processes, can provide a competitive endogenous RNA mechanism. circRNAs can function as sponges which bind directly to miRNAs, resulting in the post-transcriptional regulation of downstream targets [8]. Our group also made some researches on the interaction between circRNA and miRNA, we found that circAFAP1 could serve as sponges to miR-374b-3p and thus promotes clear cell renal cell carcinoma growth and angiogenesis [9] circRNAs appear to have a variety of functions in the onset and progression of prostate cancer, according to several studies. CircRNA-51217 may sponge miRNA-646 and boost TGF1 production, inducing TGF1/P-SMAD signalling and thereby increasing prostate cancer cell invasion [10]. The hsa-circ-0005276/FUS axis upregulates XIAP, which promotes prostate cancer cell proliferation, migration, and epithelial-mesenchymal transformation [11]. As sequencing technology advances, it is expected that additional undefined circRNAs and their roles in prostate cancer will be found.

In the present study, we identified a circRNA, termed circDHRS3 (or called hsa_circ_0010023), the function of which is to inhibit prostate cancer progression. CircDHRS3 was a previously unknown circRNA transcribed from human DHRS3 (dehydrogenase/reductase 3) gene. Has_circ_0010023 is formed by back-splicing of the linear transcript of DHRS3 gene with a length of 359 nucleotides. CircDHRS3 was found to be related to the Gleason grade of prostate cancer and might be a potential biomarker for further diagnosis of the malignant degree of prostate cancer. *In vitro* and *in vivo* overexpression of circDHRS3 has been shown to decrease prostate cancer growth and migration. We also found that the circDHRS3/miR-421/MEIS2 axis may play a role in the onset and progression of prostate cancer. Thus, circDHRS3 has the potential to be a prostate cancer biomarker and therapeutic target.

## Materials and methods

### Patient samples and circRNA sequencing

Prostate cancer tissues were taken from a radical prostatectomy at Huashan Hospital, Fudan University [12], while benign prostate hyperplasia tissues were collected via transurethral resection of the prostate. For future investigation, all tissues were stored in liquid nitrogen or 4% paraformaldehyde, whereby two pathologists examined them histologically to confirm the diagnosis. Each participant signed a written informed consent form. Fudan University’s Huashan Hospital’s Board of Directors and Ethics Committee authorized the initiative (KY2011-009). RNase R was used to extract total RNA, which was subsequently subjected to high-throughput sequencing. circRNA was subjected to a differential gene expression study [13]. The differentially expressed circRNAs were defined as those with *p* < 0.05 and |log2FC| > 1.

### Cell lines

The RWPE-1, BPH-1 and prostate cancer (22Rv1, PC3, Du145) cell lines were purchased from the Type Culture Collection at the Chinese Academy of Sciences (Shanghai, China). K-SFM medium (Gibco) was used to cultivate RWPE-1. Dulbecco’s modified Eagle’s medium (Gibco) was used to cultivate PC3 and Du145 cells. Using a 1640 medium (Gibco), 22Rv1 cells were incubated. All cells were grown in media containing 10% foetal bovine serum, 100 units/mL penicillin, and 100 mg/mL of streptomycin and kept in a humidified incubator with 5% CO_2_.

### Quantitative reverse transcriptase PCR (qRT-PCR)

TRIzol reagent was used to extract total RNA from tissues or cells according to the manufacturer’s instructions (Thermo Fisher Scientific, Invitrogen).

### Cell transfection and vector construction

shRNA, siRNA, and miRNA mimics and inhibitors (GenePharma, Shanghai, China) were transfected into cells using Lipofectamine 2000 (Invitrogen, USA). All primers and oligo sequences are provided in the supplemental material [1415^15^.

### *RNA fluorescence* in situ *hybridization (FISH)*

Specific circDHRS3 and miR-421 probes were produced (Geneseed Biotech, Guangzhou, China), and the FISH experiment was done as reported before.

### RIP assay

The Magnetic RIP RNA-binding protein immunoprecipitation kit was used for the RIP experiment (Millipore) [14].

### RNA pulldown

The incubation of biotin-labelled circDHRS3 or oligo probes with streptavidin-coupled magnetic beads (Life Technologies, USA) was used to make probe coated beads, which were subsequently used for the RNA pulldown analysis [14].

### Western blotting analysis

As in our previous study, the protein of prostate cancer cells or tissues was collected from cells lysed in an ice-cold RIPA lysis buffer and was separated on SDS-PAGE to measure the relative protein expression [14].

### Cell Counting Kit-8 assay

The Cell Counting Kit-8 assay (Sigma-Aldrich) was used to measure cell proliferation. Cell viability was measured at 0, 24, 48, and 72 h after the cells were seeded into 96-well plates.

### Colony formation assay

Five hundred cells per well were seeded into a 6-well plate. After 8 days of growing in complete DMEM, the colonies were fixed with 4% paraformaldehyde and stained with crystal violet.

### Transwell assay

Transwell chambers (Co-Star) were used to measure cell invasion according to the manufacturer’s instructions. 150 μL of cell suspension containing approximately 40,000 cells were added to the Transwell chamber, and the medium used to resuspend the cells does not contain foetal bovine serum. The lower chamber was added with 600 μL of complete medium containing FBS. Cells were cultured under conventional conditions for 24 hours. Remove the chambers, discard the medium from the upper and lower chambers, and wash the upper and lower chambers twice with PBS. Wipe off the cells in the upper chamber with a cotton swab. Add 4% PFA solution to fix for 15 min and wash the chambers twice with PBS. The chambers were stained with crystalline violet staining solution for 10 minutes and washed for 3 times with PBS. The results of Transwell assay were imaged in bright field under an inverted phase contrast microscope and analysed.

### Wound-healing assay

A straight scratch was formed after seeding 3 × 10^4^ PC3 or Du145 cells per well into a 6-well plate. Mitomycin C (5 ug/ml) in full DMEM was used to stop cell growth. The wounds were assessed 24 h after they were scratched.

### Luciferase reporter assay

To perform the dual-luciferase reporter assay, wild-type and mutated circDHRS3 and MEIS2 3’-UTR cDNA fragments were cloned and recombined into psiCHECK-2 (Promega, Madison, WI, USA) as previously described [16].

### Animal study

BALB/c Nude mice (6 weeks old, male) were purchased from SLACOM (Shanghai, China) and then were used as xenograft hosts.

A total of 1 × 10^7^ PC3 cells transfected with LV-NC and LV-circDHRS3 were implanted subcutaneously in nude mice, which were then monitored for 5 days. The mice were euthanized after 21 days, and tumours were removed and weighed. For the metastasis investigation, PC3 cells were transfected with Luc expression vectors into LV-NC and LV-circDHRS3. Then, the cells were injected intravenously (2 × 10^5^) and intratibially (2 × 10^7^) into the tails of three randomly selected mice, respectively. Bioluminescence imaging was used to investigate PC3 cell metastasis 30 days post the intravenous injection of luciferin (150 mg/kg body weight) into the mouse tails.

### Bioinformatic analysis

CircBank, Encori, Circinteractome, MiRanda, TargetScan, miRWalk, and TCGA were applied to investigate the interaction of circRNA/miRNA/mRNA. Based on MEIS2 expression, patients in the TCGA-PRAD study were classified into high and low groups.

## Results

### CircDHRS3 is down-regulated in high-grade prostate cancer

**Figure 1.**
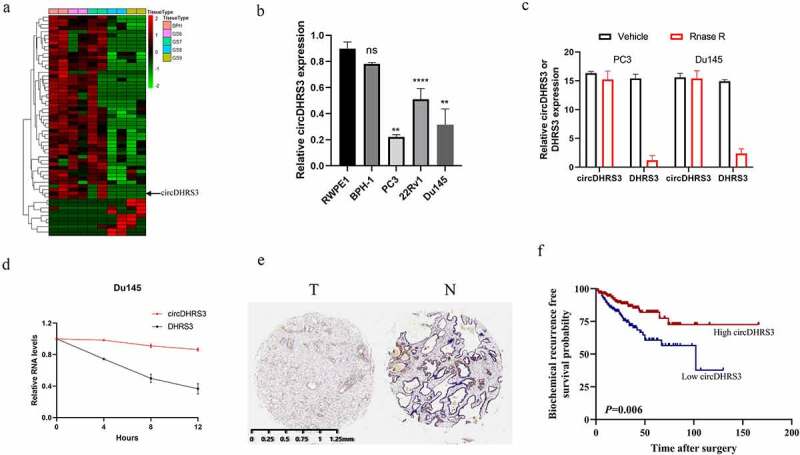
**circDHRS3 is down-regulated in high-grade prostate cancer**. (a). Cluster heatmap for circRNAs among various pathological diagnoses (filtered by *p* < 0.05 and |log2FC| > 1). Columns show tissues while rows show differentially expressed circRNAs, with circDHRS3 identified on the right of rows. (b). qRT-PCR was used to look at the expression of circDHRS3 in normal prostate epithelial cells RWPE-1 and prostate cancer cell lines 22Rv1, PC3, and Du145. The data were presented as means ± SD. (***p < 0.001, **p < 0.01 versus RWPE-1). (c). Expression of circDHRS3 and linear mRNA-DHRS3 either treated or left untreated with RNase R as detected by qRT-PCR. circDHRS3 is more resistant to RNase R than mRNA. Data are presented as the mean ± SD. (d). qRT-PCR detected the residual level of circDHRS3 and linear mRNA-DHRS3 in Du145 cells treated with Actinomycin D at the specific time point. Data were shown as the means ± SD. (e). Tissue microarray data in prostate cancer tissues compared to precancerous prostate tissues by ISH revealed that circDHRS3 is downregulated in prostate cancer tissues (Left panel) compared to paracancerous prostate tissue (Right panel). (f). Survival curves of patients with prostate cancer of high or low expression of circDHRS3, according to the ISH, using the median value as the grouping criterion. The follow-up time spans 7 years after surgery.

We extracted total RNA from two paired prostate cancer of different Gleason grade prostate tissues and then analysed the differential circRNAs between each tissue to assess for any potential correlations between circRNAs and different Gleason grades in prostate cancer. Between paired prostate cancer tissues of various pathological diagnoses, 10 upregulated and 232 downregulated circRNAs were found (*p* < 0.05 and |log2FC| > 1). circDHRS3, with a length of 359 bp sourced from (Sanger) sequencing using the unique back-splicing site of circDHRS3 as opposed to DHRS3 (Figure 1a), appeared to be downregulated in prostate cancer of high Gleason grade. Afterwards, the expression of circDHRS3 was tested in both healthy (RWPE-1) and cancerous (22Rv1, Du145, and PC3) prostate epithelial cells by qRT-PCR. The results of the qRT-PCR showed that the expression of circDHRS3 in the Du145 and PC3 cell lines was decreased in comparison to RWPE-1 (Figure 1b). As suspected, circDHRS3 was more resistant to RNase R digestion than its linear RNA (Figure 1c) and was more stable under the Actinomycin D treatment than its linear counterpart (Figure 1d). Furthermore, using *in situ* hybridization (ISH) on tissue microarrays, we found that circDHRS3 was downregulated in prostate cancer tissues when compared to precancerous prostate tissues (Figure 1e). We then separated the samples into two groups based on their ISH staining levels, using the median value as the grouping criterion. A Kaplan–Meier plot revealed that the high expression group (n = 185) had a poorer prognosis than that of the low expression group (n = 184) (figure 1f), suggesting that circDHRS3 might be used as a marker to predict prostate cancer prognosis. Summarizing the above two points, we indicated that the circDHRS3 transcript comprises a circular formation. The expression of circDHRS3 was linked to prostate cancer Gleason grades, prompting us to investigate the underlying mechanism of circDHRS3ʹs participation in distinct Gleason grades of prostate cancer.

### Overexpression of circDHRS3 decreased the proliferation and metastasis of prostate cancer cell lines in vitro

**Figure 2.**
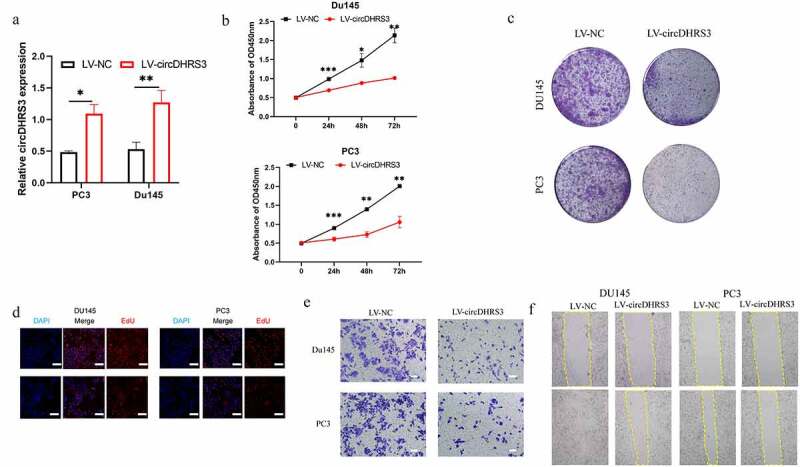
**Overexpression of circDHRS3 decreased the proliferation and metastasis of prostate cancer cell lines in vitro**. (a). qRT-PCR was used to determine the efficacy of LV-circDHRS3 overexpression in Du145 and PC3 cells, compared to the negative control (NC). Data are presented as the mean ± SD. (b). CCK-8 proliferation assays of Du145 and PC3 were detected to perform the proliferation of LV-circDHRS3 and NC. Data are presented as the mean ± SD. (c). A colony formation experiment was used to determine the ability of Du145 and PC3 cells transfected with LV-circDHRS3 and LV-NC to produce colonies. (d). The proliferation ability of Du145 and PC3 cells transfected with LV-circDHRS3, and LV-NC was assessed using an EdU assay. (Scale bars = 50 μm). (e). Invasion ability of Du145 and PC3 cells transfected with LV-circDHRS3, and LV-NC was determined using transwell assays. (Scale bars = 100 μm). (f). Wound healing was applied to assess the migration ability of Du145 and PC3 cells transfected with LV-circDHRS3 and LV-NC.

To further explore the role of cirDHRS3 in prostate cancer of various Gleason grades, a lentiviral over-expression vector was created (LV-circDHRS3). According to the results of the qRT-PCR analysis, LV-circDHRS3 can boost circDHRS3 expression in Du145 and PC3 cell lines (Figure 2a). Then, the proliferation of Du145 and PC3 cell lines were inhibited due to the over-expression of circDHRS3 (LV-circDHRS3) by the CCK8 (Figure 2b), colony formation (Figure 2c), and EdU assays (Figure 2d). Additionally, transwell assays indicated that the over-expression of circDHRS3 inhibited the migration ability of Du145 and PC3 cell lines (Figure 2e). Furthermore, wound healing assays showed that the LV-circDHRS3 of Du145 and PC3 cell lines had a more propensity to migrate than the LV-NC Du145 and PC3 cell lines (figure 2f).

### CircDHRS3 inhibits the expression of miRNA and serves as an RNA sponge for miR-421

**Figure 3.**
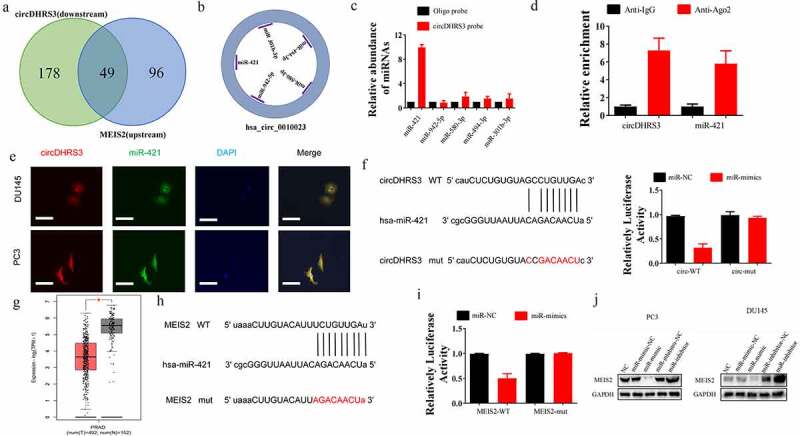
**circDHRS3 inhibits the expression of miRNA and serves as an RNA sponge for miR-421 and miR-421 serves as an mRNA inhibitor to downregulate MEIS2 expression**. (a and b). Venn diagram of the predicted downstream targets of circDHRS3 and upstream targets of MEIS2 from Encori and software circMIR, shown as a schematic illustration. (c). The affinity of circDHRS3 for miR-421 in PC3 cells was assessed using RNA pull-down. qRT-PCR detection of relative miRNA expression under the enriched circDHRS3 probe. Data are presented as mean ± SD. (d). RIP experiments were performed in PC3 cells against anti-immunoglobulin G or Ago2, and the precipitated circDHRS3 and miR-421 were detected by qRT-PCR. (e). FISH showed the subcellular co-localization between circDHRS3(red) and miR-421(green). DAPI (blue) was used to stain the nucleus. (Scale bars = 25 μm). (f). Dual-Luc reporter assays show the binding properties of circDHRS3 and miR-421. The Mut version of circDHRS3 is also shown. (g). The GEPIA database showed MEIS2 down-expression in prostate cancer tissue versus normal tissue. *p < 0.05. (h). Dual-Luc reporter assays showed binding properties of miR-421 and MEIS2. The Mut version of the 3ʹUTRMEIS2 is also shown. (i). Relative Luc activity was determined 2 days after transfection with the miR-421 mimic/normal control or with the 3ʹUTR-MEIS2 WT/Mut in PC3 cells. Data are presented as the mean ± SD. (j). Western blot displayed the MEIS2 levels among PC3 and Du145 cells, both of which were transfected with miR-NC, miR-mimic, miR-inhibitor-NC, or miR-inhibitor, respectively. GAPDH was used as the reference gene.

We used bioinformatics to analyse and predict the downstream targets of circRNAs. A literature survey revealed that MEIS2 is downregulated in metastatic sites of prostate cancer [17]. Both Venn and predicting diagrams suggested the potential miRNA, which was predicted by Encori (Figure 3a and b). miR-421 was found to participate in the ADT resistance, Enzalutamide resistance [18], and Kazal type-1 (SPINK1)-positive prostate cancer, the second most recurring and aggressive subtype of prostate cancer [19]. Thus, we chose miR-421 as a downstream proxy of circDHRS3. The strongest interaction between circDHRS3 and miR-421 was found using an RNA pull-down assay to further clarify the true downstream target of circDHRS3 (Figure 3c). The association between circDHRS3 and miR-421 was then confirmed using Ago2 RNA immunoprecipitation (RIP). Results indicated that higher circDHRS3 and miR-421 levels were found in anti-Ago2 RIP than those in anti-immunoglobulin G RIP (Figure 3d). The results of FISH revealed that circDHRS3 and miR-421 were co-localized in the cytoplasm (Figure 3e). We designed dual-luciferase reporter vectors that contained wild-type (WT) and mutated (Mut) circDHRS3 to be cloned and co-transfected with miR-421 mimics or with miR-NC into Du145 cell lines (figure 3f). Dual-luciferase assays indicated that the co-transfected WT reporter vector and miR-421 mimics suppressed luciferase activity significantly, but Mut did not. Based on the latter results, we pointed out that miR-421 is the downstream target of circDHRS3 and may play a role as a prostate cancer promoter.

Next, we assessed whether the downstream target of miR-421 is MEIS2. We further summarized the expression of MEIS2 among 492 prostate cancer and 152 control samples using the GEPIA database (http://gepia2.cancer-pku.cn/). We found that MEIS2 was down-regulated in prostate cancer samples (Figure 3g). Further, we conducted a dual-luciferase assay, from which we found that the luciferase activity of WT MEIS2 mRNA sequence transfected cells was decreased significantly after the transfection miR-421 mimics. However, the luciferase activity of Mut MEIS2 sequence transfected cells remained unchanged (Figure 3h and i). Additionally, the western blot analysis was used in PC3 and Du145 cell lines, and the results suggested that miR-421 could down-regulate the expression of MEIS2 while miR-421 inhibition could up-regulate the expression of MEIS2 (Figure 3j).

1. **miR-421 overexpression or MEIS2 silencing restored proliferation, migration, and invasion of prostate cancer cells post circDHRS3 overexpression**

**Figure 4.**
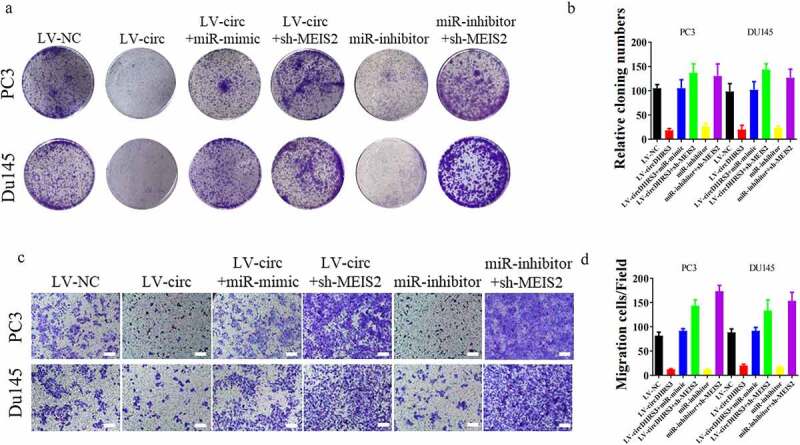
**miR-421 overexpression or MEIS2 silencing restored proliferation, migration, and invasion after circDHRS3 overexpression**. (a and b). Colony formation assays were performed to detect the colony formation ability of Du145 and PC3 cells transfected with LV-NC, LV-circDHRS3, LV-circDHRS3+ miR-mimic, LV-circDHRS3+ sh-MEIS2, miR-inhibitor, and miR-inhibitor + sh-MEIS2. Data are presented as the mean ± SD. (c and d). Transwell assays were performed to detect the colony formation ability of Du145 and PC3 cells transfected with LV-NC, LV-circDHRS3, LV-circDHRS3+ miR-mimic, LV-circDHRS3+ sh-MEIS2, miR-inhibitor, and miR-inhibitor + sh-MEIS2. Data are presented as the mean ± SD. (Scale bars = 100 μm).

**Figure 5.**
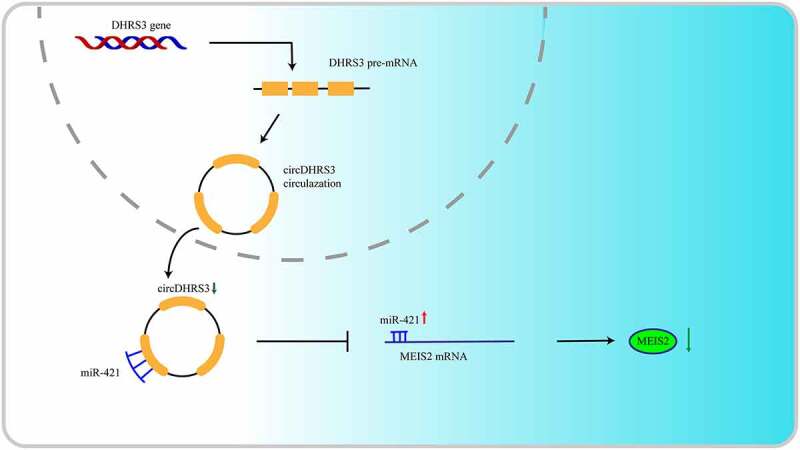
Schematic diagram of circDHRS3/MiR-421/MEIS2 pathway in PCa.

We established PC3 and Du145 cell lines transfected with MEIS2 knockdown or overexpressed miR-421 to further uncover the relevance of the circDHRS3/miR-421/MEIS2 axis in regulating the proliferation, migration, and invasion of prostate cancer. Next, colony formation assays were performed to investigate the role of cell proliferation *in vitro*. The experimental results illustrated that the inhibition of MEIS2 or the overexpression of miR-421 promoted the proliferation of circDHRS3 overexpressed PC3 and Du145 cell lines. In contrast, silencing miR-421 inhibited the proliferation of PC3 and Du145 cell lines, which could be reversed by transfection with shMEIS2 (Figure 4a and b). By contrast, the transwell assays revealed that the overexpression of circDHRS3 decreased the abilities of migration and invasion of the PC3 and Du145 cell lines, however, by silencing the MEIS2 gene or by up-regulating miR-421 reversed this phenomenon. The inhibition of miR-421 also reduced this ability, but the addition of shMEIS2 reversed the abilities of migration and invasion (Figure 4c and d). A schematic diagram of circDHRS3/MiR-421/MEIS2 pathway in PCa was shown in Figure 5.

### *Overexpression of circDHRS3 decreased prostate cancer proliferation and metastases* in vivo

**Figure 6.**
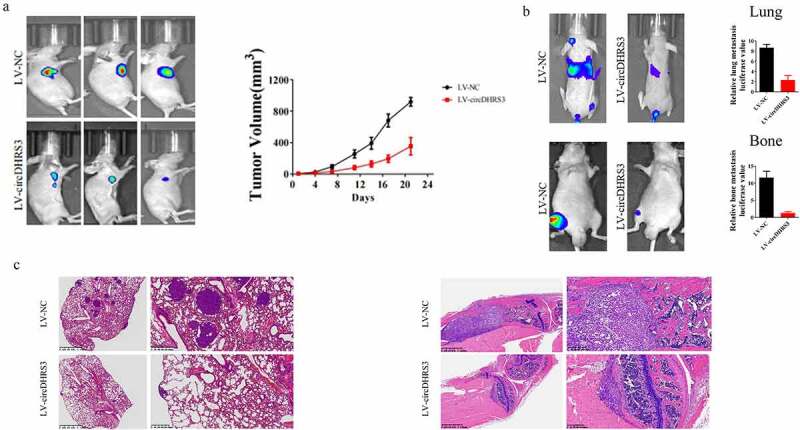
**Overexpression of circDHRS3 decreased prostate cancer proliferation and metastases in vivo**. (a). Tumour sizes were measured and compared for LV-NC-PC3 and LV-circDHRS3-PC3 xenografts in nude mice after 21 days. Tumour formation was detected using live imaging. (b). The lung or bone metastasis ability of LV-NC-PC3-Luc and LV-circDHRS3-Luc by intravenous tail injection or intratibial injection was detected using live imaging. (c). HE staining of lung and bone after injection of LV-NC-PC3-Luc and LV-circDHRS3-Luc.

A subcutaneous tumour formation assay was conducted to explore the relationship between circDHRS3 and prostate cancer *in vivo*. While circDHRS3 was overexpressed, the subcutaneous tumour size of the LV-circDHRS3 group was smaller than that of the LV-NC group. The difference in tumour volume increased over time (Figure 6a). Furthermore, we injected luciferase (Luc)-labelled PC3 cells containing the overexpression of circDHRS3 (LV-circDHRS3) and LV-NC into mice via the tail vein or tibia separately. After 21 days, *in vivo* bioluminescence images were taken, the results of which showed that the LV-circDHRS3 group exhibited a reduction in fluorescence MONG lung and bone metastases sites (Figure 6b). HE staining further corroborated that circDHRS3 overexpression decreased prostate cancer metastases (Figure 6c).

## Discussion

circRNAs have recently been identified to play a role in the occurrence and progression of cancer; however, further investigations and trials are required before applying them in a clinical setting. Our teammates did a research and found that circUBE2K could mediate RhoA associated bladder cancer phenotype via regulation of miR-516b-5p/ARHGAP5 axis [20]. The upregulation of hsa_circ_0003258 promotes tumour formation via the hsa_circ_0003258/miR 653p/ARHGAP5 axis, as well as the hsa_circ_0003258/IGF2BP3/HDAC4 axis, according to a recent study [21]. Hsa_ circ_0003258 could be a useful biomarker for prostate cancer metastasis and a potential therapeutic target. As stated in a previous review, accurate analysis and detection are required for the use of circRNA as a biomarker [22], although the circRNA detection technology has limitations, particularly in terms of sensitivity and stability [22].

In our discovery of the relationship of circRNAs and prostate cancer, we explored a novel circRNA lowly expressed in prostate cancer, namely circDHRS3. circDHRS3 was significantly related to Gleason grades. The overexpression of circDHRS3 could inhibit prostate cancer cell proliferation and metastases both *in vitro* and *in vivo*. Otherwise, circDHRS3 serves as an RNA sponge of miR-421. circDHRS3 could bind directly to miR-421 in an AGO-dependent manner. Both the bio information forecast and the dual-luciferase verification suggested that 3′ UTR of circDHRS3 and MEIS2 shared the complementary miR-421 binding sequence. The rescue assay further indicated that circDHRS3 could regulate MEIS2 expression in prostate cancer by antagonizing miR-421.

As a transcription factor, MEIS2 plays a key role in determining cell fate during development and proliferation. MEIS proteins bind and direct HOX protein transcriptional specificity, which is one of their primary functions [23,24]. It has been shown that MEIS1 and MEIS2 expression is associated with a more indolent phenotype of prostate cancer, with a lower risk of metastatic development [17]. Additionally, MEIS2 expression is decreased in poor-prognosis tumours [25] and participates in the emergence of castration-resistant prostate cancer [26]. In our study, knockdown MEIS2 expression may promote the invasion of prostate cancer.

In summary, circDHSRS3 was discovered as a Gleason grade related to circRNA. circDHRS3 is a miRNA sponge for miR-421 which targets the expression of MEIS2 to inhibit the proliferation and invasion of the prostate cancer cell. The circDHRS3/miR-431/MEIS2 axis could influence the occurrence and development of prostate cancer. MiR-421 and MEIS2 are both correlated with prostate cancer and ADT resistance, but whether this axis influences ADT resistance remains unknown.

## Supplementary Material

Supplemental Material

## Data Availability

On reasonable request, the corresponding author can provide the datasets used and/or analyzed during this investigation. The data that support the findings of this study are available from the corresponding author, Haowen Jiang(haowj_sh@fudan.edu.cn), upon reasonable request.

## List of abbreviation

**ADT**: Androgen deprivation therapy; **CCK-8**: Cell Counting Kit-8; **circRNA**: Circular RNA; **FISH**: Fluorescence *in situ* hybridization; **ISH**: *in situ* hybridization; **NC**: Negative control; **qPCR**: Quantitative reverse transcriptase PCR; **RIP**: RNA Binding Protein Immunoprecipitation; **SD**: Standard Deviation; **TCGA**: The cancer genome atlas; **WB**: Western blot; **WT**: wild-type
